# Effect of Silica-Modified Aluminum Oxide Abrasion on Adhesion to Dentin, Using Total-Etch and Self-Etch Systems

**DOI:** 10.3390/polym15020446

**Published:** 2023-01-14

**Authors:** Shifra Levartovsky, Benny Ferdman, Nahawand Safadi, Tujan Hanna, Eran Dolev, Raphael Pilo

**Affiliations:** 1 Department of Oral Rehabilitation, The Maurice and Gabriela Goldschleger School of Dental Medicine, Tel Aviv University, Tel Aviv 6997801, Israel; 2 The Maurice and Gabriela Goldschleger School of Dental Medicine, Sackler Faculty of Medicine, Tel Aviv University, Tel Aviv 6997801, Israel; 3 Department of Oral Biology, The Maurice and Gabriela Goldschleger School of Dental Medicine, Sackler Faculty of Medicine, Tel Aviv University, Tel Aviv 6997801, Israel

**Keywords:** shear bond strength, total-etch, self-etch, air abrasion, dentin

## Abstract

This study compared the shear bond strength (SBS) and micromorphology of composite resin to human dentin after pre-treatment with silica-modified aluminum oxide air abrasion. Forty-six molar teeth were treated with either Scotchbond Multi-Purpose (SCMP) or Clearfil SE Bond (CLSE) adhesive. Buccal surfaces were pre-treated with the CoJet air abrasion system (SB), and lingual surfaces were controls. The adhesion of light-cured resin composite to the treated dentin surface was evaluated with SBS. After debonding, substrate surfaces were examined with an optical microscope for failure analysis. In addition, 15 molar teeth were sectioned and randomly assigned to one of five groups, according to the dentin surface pre-treatment and adhesive type, and examined with high-vacuum scanning electron microscopy/energy dispersive X-rays (SEM/EDS). The type of adhesive had a significant effect on SBS (*p* = 0.000); CLSE had the highest values. SB did not affect SBS (*p* = 0.090). SEM/EDS revealed residual aluminum and/or silicon on all dentin surfaces after SB, except for the control. Treatment with 32% phosphoric acid in the SCMP adhesive decreased the amounts of aluminum and silicon compared to SB dentin only, whereas CLSE resulted in similar quantities of aluminum and silicon as air-abraded dentin. The results of this study indicate that CLSE might have a higher bond strength to dentin than SCMP. Pre-treatment with SB does not appear to affect bonding strength.

## 1. Introduction

Dentin bonding has become one of the most challenging procedures in restorative dentistry, due to the organic content and tubular structure of the dentin, with its fluid flow in an outward direction and the smear layer covering the cut dentin surface [1].

Current adhesive systems that interact with the dentin substrate either remove the smear layer completely (total-etch technique, TE) or maintain it as the substrate for the bonding (self-etch technique, SE) [2]. Unlike TE adhesives, SE adhesives do not completely resolve or remove the smear layer, but rather partly integrate it into the hybrid layer [3]. SE has become attractive due to reduced technique sensitivity of the bonding procedure, lower risk of resin incompletely impregnating the demineralized dentin, and less post-operative sensitivity [4,5].

Several studies demonstrated that SE adhesives can provide adhesion to dentin that is comparable or even superior to that of TE adhesive systems [4,5,6]. Although the mechanism is still unclear, it is assumed that the penetration of the SE monomers into the dentin beyond the hybrid layer and the chemical interaction between the functional monomer and hydroxyapatite may contribute to the formation of an acid–base-resistant zone beneath the hybrid layer, which contributes to the resin–dentin bonding strength [7].

Pre-treating the dentin surface using airborne abrasion with aluminum oxide was found to increase the surface area available for adhesion, enhance resin tag formation, and improve bond strength to dentin [8,9]. As the aluminum oxide particles collide with dentin, their kinetic energy is released, which fractures microscopic fragments and creates a rough tooth surface that is more conducive to bonding [10,11]. Previous research has reported negative and positive effects, as well as no effect, of air abrasion on the bond strength to dentin. Freeman et al. studied the effect of air abrasion and thermocycling on the adaptation and shear bond strength (SBS) of composite resin bonded to dentin using TE and SE resin adhesives [12]. They found that, even though air abrasion tended to increase the number, length, and diameter of resin tags, especially in the SE adhesive, it also increased the number of defects observed in the hybrid layer on the dentin surface, and therefore, only minimally enhanced the SBS of resin to dentin. Anja et al. compared the micro tensile bond strength of SE adhesive to human dentin surface modified with air abrasion and sonic techniques and found no difference in the micro tensile bond strength between the control (no pre-treatment) and the air abrasion and sonic preparations [11]. Similarly, Franca reported that previous dentinal air abrasion with aluminum oxide did not alter the bond strength of SE adhesive systems at various evaluation times [13]. On the other hand, Sutil et al. evaluated the effects of dentin pre-treatment and temperature on the bond strength of a Scotchbond Universal Adhesive system to dentin, in SE versus TE mode. They found that dentin surface treatment with sodium bicarbonate air abrasion improved the bond strength, regardless of the adhesive application mode [14].

The current study compared the SBS and micromorphology of composite to human dentin surface, after pre-treatment with aluminum oxide air abrasion, using TE versus SE adhesive systems. The null hypotheses were as follows: (1) No significant differences would be found in the SBS between the two adhesive systems. (2) No significant differences would be found between the untreated and treated dentin surfaces.

## 2. Materials and Methods

### 2.1. Sample Preparation

**Figure 1:**
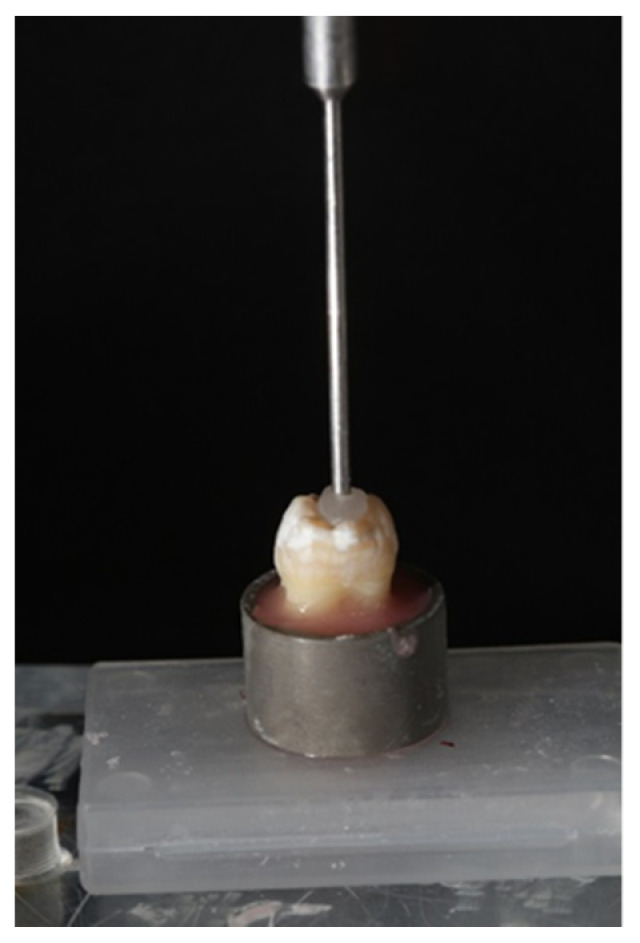
Sample preparation: Teeth were suspended in the center of an aluminum cylinder and mounted 2 mm apical to the cement–enamel junction in polymethyl methacrylate resin.

The study sample included 46 freshly extracted, caries-free, intact molars with similar dimensions, obtained from individuals aged 30–50 years. After all external debris was removed with curettes, the teeth were stored in a germ-free, 0.1% thymol solution (Sigma-Aldrich, Rehovot, Israel) at room temperature. Each tooth was embedded parallel to the long axis of the tooth with a custom-designed alignment apparatus in the center of an aluminum cylinder (20 × 25 mm) and mounted 2 mm apical to the cement–enamel junction in polymethyl methacrylate resin (Quick Resin, Ivoclar Vivadent, Schaan, Liechtenstein) [15] (Figure 1).

To expose a flat dentin surface, the superficial lingual and buccal enamel were removed using a rigidly secured, high-speed grinder equipped with a diamond burr, under air–water irrigation (F2—coarse followed by fine; Strauss, Ra’anana, Israel). The high-speed grinder was mounted on a custom-designed, surveyor-like apparatus (6.5° taper) and a new burr was used for each tooth [15].

#### SBS Test Samples

The 46 prepared teeth (92 buccal and lingual surfaces) were randomly assigned to two groups (2 × 23) according to the adhesive used: Scotchbond Multi-Purpose (SCMP) Dental Adhesive System (3M Dental Products, St. Paul, MN, USA) or Clearfil SE Bond Adhesive (Kuraray Medical Inc., Tokyo, Japan) (CLSE). The adhesive systems were applied to the dentin surfaces according to the manufacturers’ instructions. The buccal surfaces of all teeth were pre-treated with an air abrasion system (Sand Blasting; SB) (CoJet; 3M Dental Products, St. Paul, MN, USA), while the lingual surfaces served as controls, with no pre-treatment. The composition of the materials used, as well as the mode of application, are described in Table 1.

The adhesive material was cured using a LED light-curing unit (ART L5-OSADA, Tel Aviv, Israel) with a light intensity of 600 mW/cm^2^. The light intensity was monitored with a curing radiometer (model 100, Demetron/Kerr, Danbury, CT, USA).

**Figure 2:**
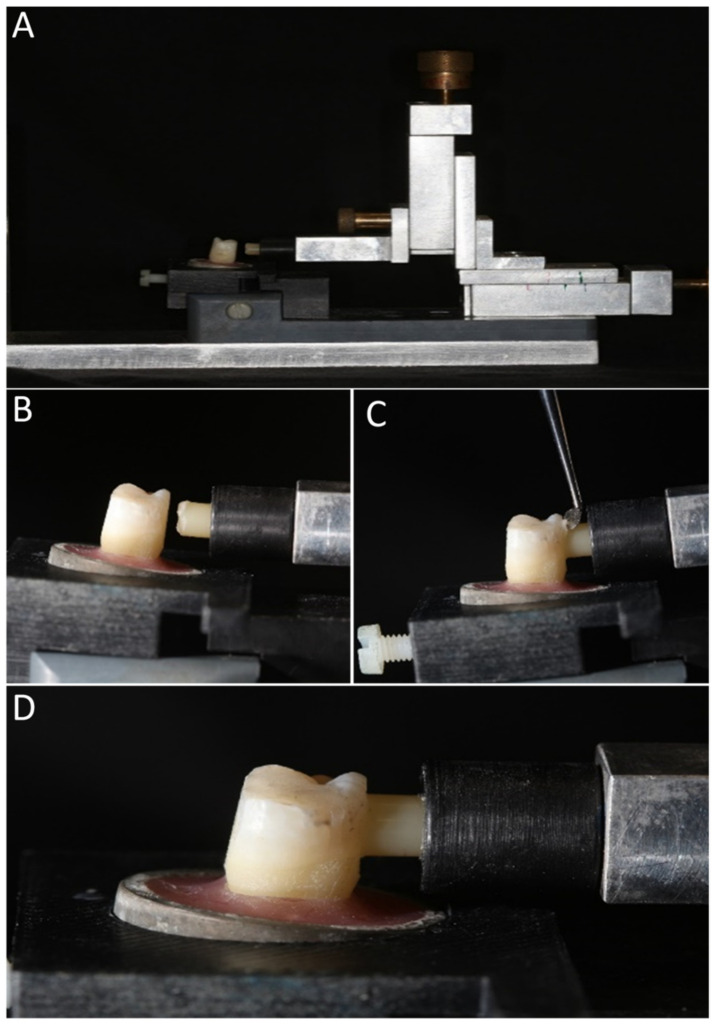
The bonding jig (**A**) and the application of the final 1 mm of composite increment perpendicular to dentin surface (**B**–**D**).

Ninety-two gelatin capsules (Torpac, Inc., Fairfield, NJ, USA) with 5 mm inner diameter and 10 mm length were filled with resin composite (Filtek Supreme XT, 3M ESPE) to 1 mm below their margins and light-cured for 40 s on each side. Resin composite was added to the remaining 1 mm of the capsule, and the surface was approximated to the adhesive-treated dentin surface. A bonding jig was fabricated to ensure perpendicular orientation of the specimens to the dentin surface (Figure 2).

### 2.2. Shear Bond Strength Test

The SBS was determined according to ISO 29022 (using the notched-head SBS test) by loading the interface of the composite cylinder and the dentin surface statically in a universal loading machine (Instron, Model 4502, Buckinghamshire, England) with a crosshead speed of 1.0 mm/min, until failure. SBS was calculated (in MPa) according to the surface area.

After testing, the debonded dentin surfaces and resin composite cylinders were examined under an optical microscope (M8 stereo microscope, Wild, Heerbugg, Switzerland) at 18× magnification. The failure modes were classified based on the criteria presented in Table 2.

For each category, the number of surfaces was summed, and the results were presented as a percentage of all surfaces exposed to the specific treatment [16].

### 2.3. Scanning Electron Microscopy (SEM) Coupled with an Energy-Dispersive Spectroscopy (EDS) Analysis

For this inspection, 15 additional freshly extracted, caries-free, intact molars were cleaned of all external debris with curettes and stored in a germ-free 0.1% thymol–tap water solution at room temperature. The teeth were sectioned perpendicular to the long axis at the level of the middle coronal third (Isomet, Plus, Buheler, Lake Bluff, IL, USA) to obtain a flat specimen of dentine, 2 mm wide, from each tooth. The specimens were randomly assigned to 5 groups (*n* = 3) according to the dentin surface treatment and the adhesive type (Table 3).

Specimens were subjected to high-vacuum scanning electron microscopy/energy dispersive X-ray microanalysis (HV-SEM/EDX). Dentin surfaces were sputter-coated with chromium in a sputter-coater unit and examined in a SEM (JSM-6700F FEG-SEM (JEOL Ltd., Tokyo, Japan) at varying magnifications (×1000–10,000) to observe the topographic patterns of the dentin. In addition, SEM coupled with energy-dispersive spectroscopy (EDS) (EDS 2000; IXRF Systems Inc., Houston, TX) was used to identify and quantify the elemental composition of the specimen areas. EDX spectra were recorded under the following conditions: 5 kV accelerating voltage, 100A beam current, 300 µm × 300 µm analysis area, 300 s acquisition time, 34% detector dead time, and 131.4 eV resolution. EDX spectra were subjected to C and ZAF (atomic number, absorption, fluorescence) corrections. Elemental analysis was performed using Genesis v 5.1 software (EDAX, Inc., Mahwah, NJ, USA).

### 2.4. Statistical Analysis

Statistical analysis for the SBS was conducted using SPSS Statistics, v27.0 (IBM Corp, Armonk, NY, USA). Two-way ANOVA was used when the dependent variable was the SBS, and the independent variables were treatment (TE or SE adhesive system) and with or without air abrasion. The data for the SBS values were further analyzed using Weibull distribution. The shape or modulus parameter (β) defines the variability of the results by expressing the size distribution of the flaws, and the scale parameter (ή), frequently defined as characteristic life, indicates the strength value at which 63.2% of the sample size will be debonded. The level of significance was set to 0.05.

## 3. Results

### 3.1. Shear Bond Strength

The SBS values in the two adhesive systems with or without air abrasion (SB) are presented in Table 4.

Two-way ANOVA revealed that the independent variable that most strongly affected the SBS was the treatment—TE or SE (*p* = 0.000), with the highest values in the Clearfil SE group. The independent variable of air abrasion (SB) did not affect the SBS (*p* = 0.09). Additionally, the interaction between SB and treatments was not significant (*p* = 0.459).

**Figure 3:**
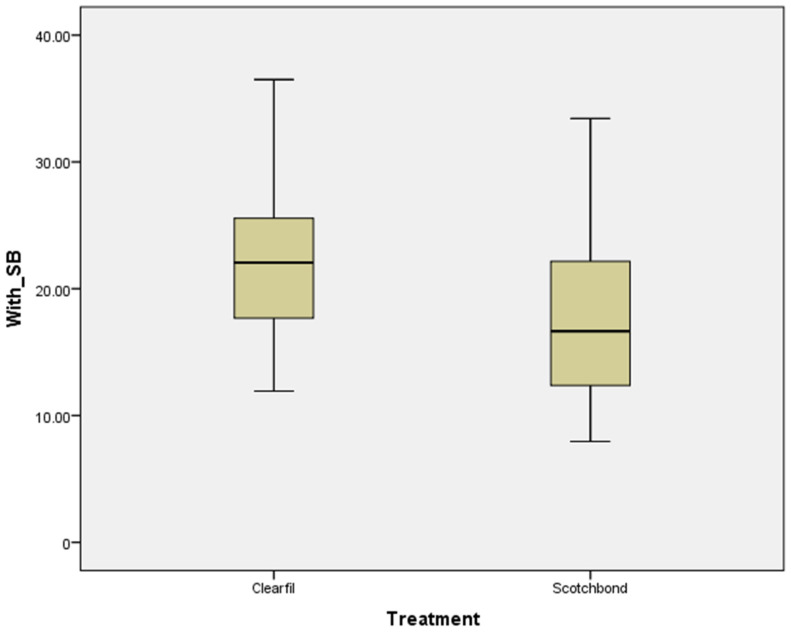
Shear bond strength (MPa) for Clearfil SE and Scotchbond Multi-Purpose with air abrasion.

**Figure 4:**
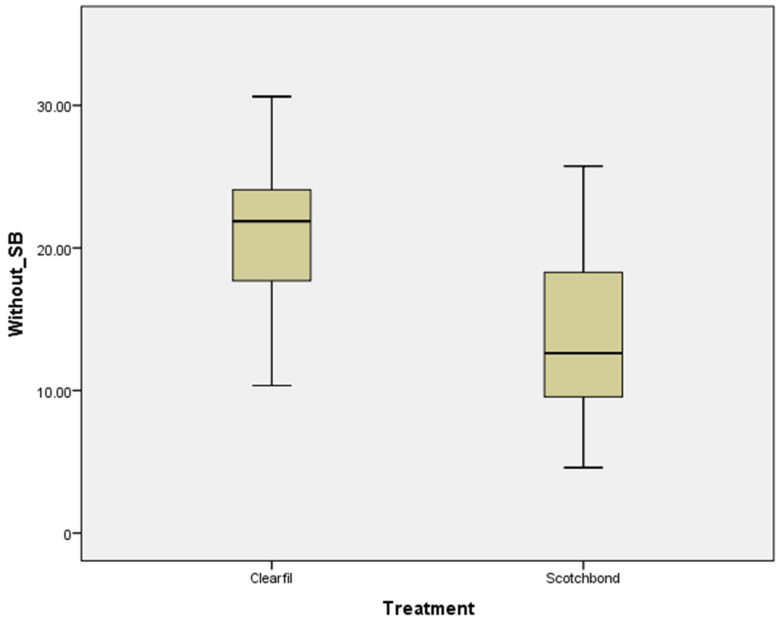
Shear bond strength (MPa) for Clearfil SE and Scotchbond Multi-Purpose without air abrasion.

Figure 3 and Figure 4 show box plot graphs of the SBS of each adhesive system (Treatment) with or without air abrasion (SB), respectively.

**Figure 5:**
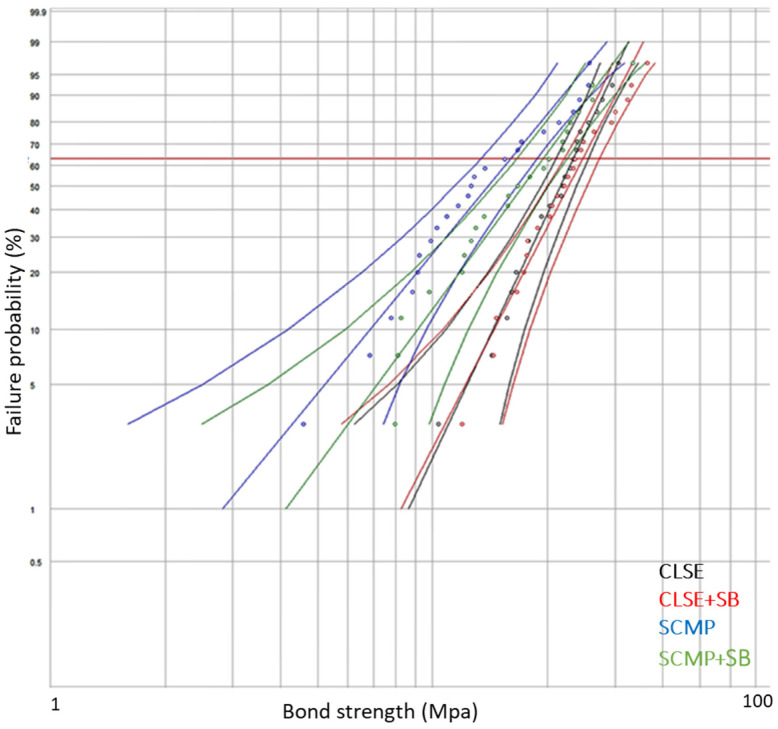
Probability plot, Weibull 95% CI. CLSE, Clearfil SE; CLSE +SB, Clearfil SE + air abrasion; SCMP, Scotchbond Multi-Purpose; SCMP + SB, Scotchbond Multi-Purpose + air abrasion.

Weibull distribution, which gave the cumulative probabilities of failure, also showed that Scotchbond Multi-Purpose (SCMP) was the weakest material regarding bonding to dentin, while Clearfil SE had the highest SBS to dentin. Weibull parameters β and ή were not significantly different regardless of air abrasion, except for the ή parameter in SCMP. The statistical difference was calculated with a 95% confidence interval (Figure 5, Table 5).

**Figure 6:**
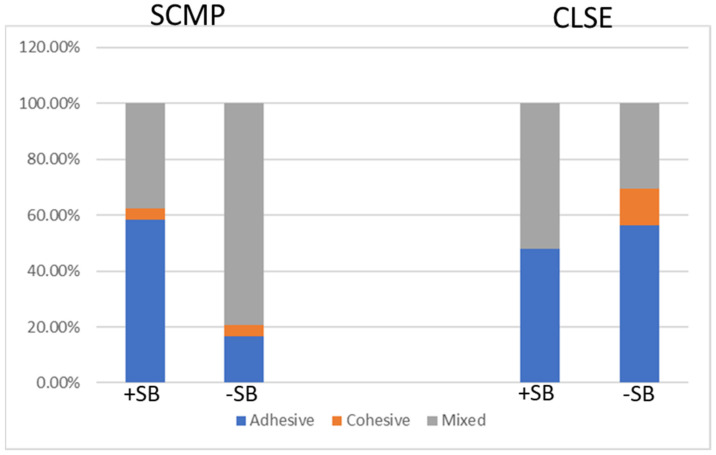
Frequencies of failure modes for the different adhesive systems. SCMP, Scotchbond Multi-Purpose; CLSE, Clearfil SE.

Examination of the failure modes after debonding revealed 58.3% adhesive failure in the SCMP with SB. In the control group, the mixed mode had the highest failure rate (79.2%). In the Clearfil SE groups, the mixed mode had the highest failure rate (52.2%) with SB, while in the control group, the adhesive mode had the most failures (56.5%) (Figure 6).

### 3.2. Scanning Electron Microscopy (SEM) Coupled with Energy-Dispersive Spectroscopy (EDS)

**Figure 7:**
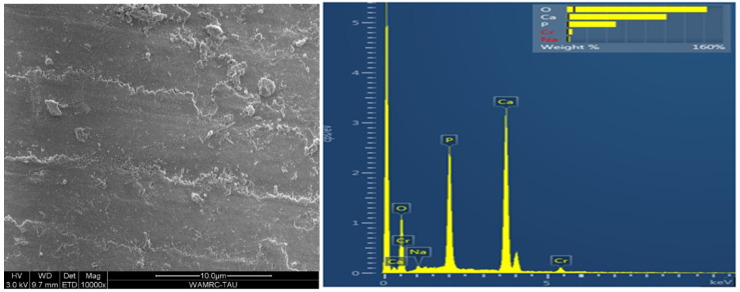
Microscopic image of the untreated control dentin. Surface is covered by smear layer, with smear plugs occluding the dentinal tubules. EDS analysis revealed the following main constituents (wt %) of intact dentin: O 48%, Ca 26.9%, P 14.1%, C 8.1%.

**Figure 8:**
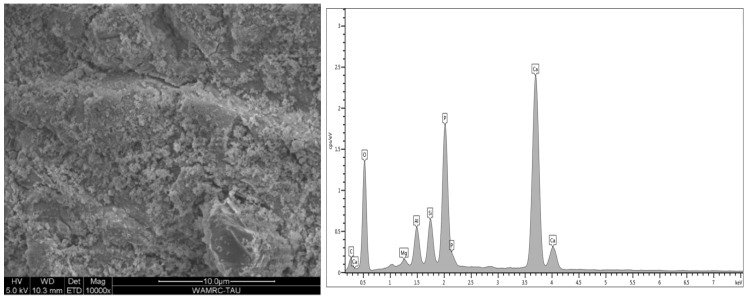
Microscopic image of the air-abraded dentin. The surface is rough and irregular. EDS analysis revealed extra elements as compared to untreated dentin (wt %): Si 5.27%, Al 2.11%.

**Figure 9:**
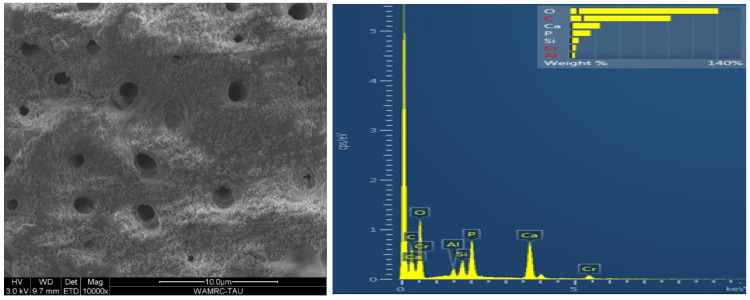
Microscopic image of the air-abraded and subsequently acid-etched dentin. The acid removed most of the smear layer and disclosed open dentinal tubules. The intertubular dentin is rough. EDS analysis revealed lower concentrations (wt %) of Si and Al, yielding 1.49% and 0.38%, respectively.

**Figure 10:**
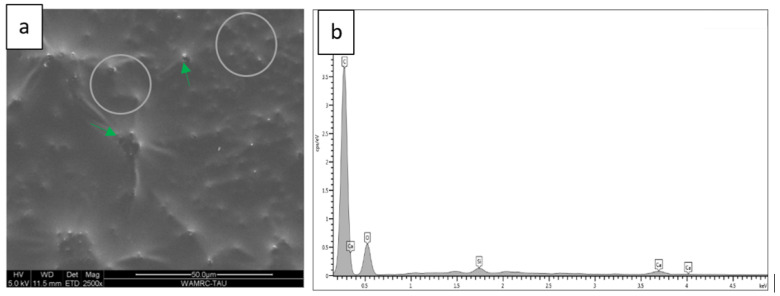
Microscopic images of air-abraded, acid-etched, and SCMP-treated dentin. The adhesive covers the surface and occludes most of the tubules (**a**). Several partly open tubules can be discerned (green arrows). White circles denoted the area of the EDS analysis, which revealed the following elements (wt %): C 63.13%, O 30.72%, Ca 2.2%, P 0.48%, Si 2.05%, Al 0.88% (**b**).

**Figure 11:**
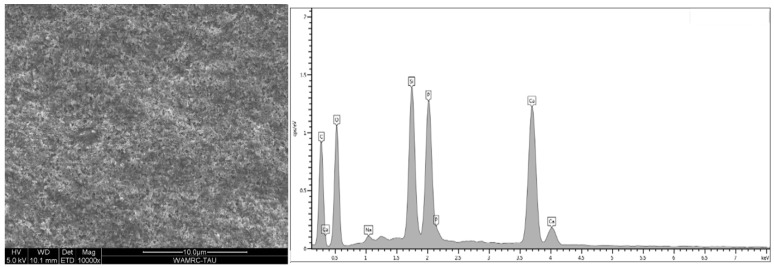
Microscopic image of air-abraded and CLSE primer- and adhesive-treated dentin. Adhesive covers the dentin surface; very few open dentinal tubules are still seen. EDS analysis revealed the following elements (wt %): C 40.98%, O 35.04%, Ca 10.51%, P 6.85%, Si 6.08%, and Al 0.1%.

Energy-dispersive analysis revealed major component elements of dentin in all test groups: carbon (C), oxygen (O), phosphorous (P), and calcium (Ca). After air abrasion, Si (silicon) and Al (aluminum) were also present (Figure 7, Figure 8, Figure 9, Figure 10 and Figure 11).

## 4. Discussion

The current study compared the shear bond strength and micromorphology of composite to human dentin surface, after pre-treatment with aluminum oxide air abrasion, using total-etch versus self-etch adhesive systems. The results led to partial rejection of the null hypothesis. The type of adhesive system affected the bond strength values, as the sixth-generation SE had significantly higher SBS than the fourth-generation TE did (~35%) and is more predictable (higher β parameter). Air abrasion (SB) did not significantly affect the SBS of the two adhesive systems.

Some of the current findings were consistent with those previously reported. Vaidyanathan and Jayalakshmi demonstrated that in SE adhesive systems, the strong electrostatic interaction between the primer monomers and hydroxyapatite, with subsequent polymerization of the monomer, promoted improved bond strength and efficient margin sealing. The interactions between the primer monomers and the collagen of the dentin have been computer-modeled and analyzed. However, in the TE adhesive system, etching the dentin with phosphoric acid may cause dehydration of the dentin and incomplete penetration of the monomers into the full depth of the demineralized region. This may leave exposed collagen fibrils, leading to voids and discontinuities in the interfacial region which facilitate nanoleakage of water into the hybrid layer [17]. Kaaden et al. compared the bond strength of SE and TE adhesives to enamel, superficial dentin, and deep dentin and found that SE had significantly higher bond strength than did TE for all three substrates [4]. Their explanation was that SE adhesives caused less damage to the dentin than phosphoric acid in the TE system did. Moreover, modification of the smear layer with the use of SE, without further demineralization of the dentin surface, might be sufficient to increase the surface energy and ensure adequate monomer diffusion, resulting in high bond strength [4].

In a review of SE versus TE adhesive systems in clinical dentistry, SE adhesive was found to provide superior and more predictable bond strength to dentin. Therefore, the authors recommended that SE is preferable for direct composite resin restorations, especially when predominantly supported by dentin, while TE bonding systems are preferred when large areas of enamel are present, since phosphoric acid creates a more pronounced and retentive etching pattern in enamel [18].

Although both TE and SE systems form a hybrid layer as a result of resins impregnating the porous enamel or dentin, Milia et al. showed in a study on extracted molars treated with either TE or SE adhesive that SE primer did not produce significant morphological changes in the moist dentin surface, while phosphoric acid severely altered the dentin collagen [19].

Among the self-etch adhesives, the mild two-step self-etch adhesive Clearfil SE is considered the gold standard because of its highly adequate dentin bonding effectiveness in vitro. Our findings, which show a superior SBS to dentin with Clearfil SE, agree with another previous report. Salvio et al. found significantly higher micro tensile bond strength with Clearfil SE Bond when compared to other SE systems and to the one-step TE system [20]. It is known that mild self-etch adhesives, such as Clearfil SE, form only submicron-thick hybrid layers, in which hydroxyapatite partially remains around exposed collagen. This residual hydroxyapatite may contribute to adhesive performance in addition to the micro-mechanical hybridization, since it serves as a receptor for chemical interactions with the functional monomer in the Clearfil SE Bond adhesive. This functional monomer is 10-methacryloxydecyl dihydrogen phosphate (10-MDP) which adheres strongly to hydroxyapatite [21]. Clearfil SE contains 10-MDP both in its primer and adhesive, while Scotchbond Multipurpose (TE) does not contain a functional monomer.

Our second null hypothesis was accepted, as the SBS of the two adhesive systems was not affected by the prior use of air abrasion. This result supported the findings of other studies. Anja et al. demonstrated that pre-treating the dentin with either air abrasion or sonic technique did not affect the bond strength of one step self-etch adhesive to human dentin [11]. Cehreli et al. did not find any differences in the effect of various techniques for removing caries, among them air abrasion, on the micro tensile bond strength to caries-affected human dentin [22]. Another study evaluated the micro tensile bond strength of a self-etch adhesive system to enamel and dentin prepared by either Er:YAG laser irradiation or air abrasion [23]. SEM analysis revealed that for both enamel and dentin, the air abrasion and laser preparations resulted in irregular adhesive interfaces, which differed from those prepared using a rotary instrument. This is clearly shown in the current study when comparing Figure 7 and Figure 8. However, Souza-Zaroni et al. reported that the micro tensile bond strengths for the test groups were similar to that of the control group. They concluded that laser energies and air abrasion tips did not increase adhesion to enamel and dentin since the surface irregularity caused by these techniques may compromise the etching ability of the SE adhesive [23].

A recent systematic review and meta-analysis evaluating the effect of airborne-particle abrasion using aluminum oxide on the bond strength of resin-based materials to dentin showed that air abrasion was favored only when the particle size was >30 μm and the pressure was >5 bar (psi = 72.5) [24]. The current study was performed with CoJet air abrasion, using 30 µm silica-coated alumina particles with 80 psi pressure; these parameters are less favorable when taking into account those advocated by Pahlavan to affect the SBS to dentin. When air abrasion with 50 μm aluminum oxide was used in another study, the shear bond strength to dentin was improved [25].

Failure analysis of the debonded specimens revealed that the dominant mode in TE with air abrasion was mainly adhesive, either between the adhesive and dentin or between the adhesive and composite, while in the control group, the predominant failure was with the mixed mode. In the SE groups, the dominant failure method with air abrasion was the mixed mode, while in the control group, the adhesive mode was the predominant failure. However, as the differences were slight, no definite conclusions can be drawn (Figure 6). Cohesive mode failures were inspected to a lesser degree and reported. It is important not to exclude these failures because omitting shear bond strengths associated with cohesive failures could bias the conclusions [26].

The micromorphology of the five dentin treatment groups, according to surface treatment and adhesive type, revealed by the SEM and EDS included remnants of residual aluminum and/or silicon on tooth surfaces after air abrasion in all groups except for the control. Treatment with 32% phosphoric acid in the TE adhesive (Figure 9 and Figure 10) decreased the amounts of aluminum and silicon as compared to air-abraded dentin only (Figure 8). On the other hand, the use of a self-etch system with mild pH resulted in similar quantities of silicon and aluminum (Figure 11) as compared to air-abraded dentin only (Figure 8). This can be explained by the 32% concentration of phosphoric acid in the TE adhesive system, which removes the smear layer completely, as well as the remnants of silicon and aluminum particles. The remnants of these particles do not interfere with adhesion, as evidenced by the comparable SBS values.

Overall, we found more silicon particles than aluminum because the CoJet air abrasion system is composed of corundum particles (a crystalline form of aluminum oxide) coated with silica. When the silica-coated aluminum particles hit the dentin surface, their kinetic energy is partially converted into thermal energy, and this increase in temperature causes the silica particles to melt and adhere to the surface, while the aluminum oxide particles fracture [27].

The limitation of this study is that clinical conditions cannot be fully reproduced in a laboratory study design. The limitations of the “macro” SBS test method which was used in the current study are as follows: stress concentration and not a pure shear state, bond strength values depend on bonding area, and the elastic modulus of the resin composite may affect test results [28]. The specimens of the dentin surface do not imitate full tooth preparation. Additionally, air abrasion was performed with CoJet, using only 30 µm silica-coated aluminum particles with 80 psi pressure, and the specimens were not artificially aged. Due to these limitations, further in vitro and in vivo studies are required.

## 5. Conclusions

Within the limitations of the current study, we can conclude that sixth-generation Clearfil SE adhesive system has a higher bond strength to dentin (~35%) compared to the fourth-generation Scotchbond Multi-Purpose adhesive system. In addition, pre-treatment using air abrasion with 30 µm silica-coated alumina particles and 80 psi pressure as a preliminary step to the bonding action does not appear to enhance or impair the bonding strength to dentin of the two adhesive systems. As such, we cannot recommend the use of air abrasion intraorally at this point due to its health risks, until research proves its usefulness.

## Figures and Tables

**Table 1:** The application protocol and composition of the adhesives and air abrasion system.

Materials	Composition	Application Protocol
Air abrasion CoJet	30 µm silica-coated aluminum oxide particles	Air abrasion at 10 mm distance for 15 s with 80 psi pressure, followed by 15 s of water spray and medium air spray.
Scotchbond Universal Etchant	32% phosphoric acid, pH 0.6	Applied to dentin for 15 s, followed by 15 s of water spray and medium air spray.
Scotchbond Multi-purpose Dental Adhesive System	Primer: Aqueous solution of HEMA, polyalkenoic acid, pH 3.3 Adhesive: Bis-GMA, HEMA, photo initiator system	Primer was applied to the etched dentin with a rubbing action for 20 s, followed by a gentle air stream for 5 s. Adhesive was then applied with a rubbing action for 20 s. Light irradiation was applied for 10 s.
Clearfil SE Bond Adhesive	Self-etch primer: 10-MDP, HEMA, hydrophilic dimethacrylate, photo-initiator, water, pH 2 Adhesive: 10-MDP, bis-GMA, HEMA, hydrophilic dimethyl acrylate, microfiller (n,n Diethanol-p-Toluidine)	Self-etch primer was applied to the etched dentin with a rubbing action for 20 s, followed by a gentle air stream for 20 s. Adhesive was then applied with a rubbing motion for 20 s, followed by a gentle air stream, and then light irradiation for 10 s.

**Table 2:** Classification of failure criteria.

Criteria	Description
Adhesive mode	Either between adhesive and dentin or between adhesive and composite
Cohesive mode	Either within the composite resin or within the adhesive layer
Mixed mode	Mix of adhesive and cohesive modes

**Table 3:** Description of study groups analyzed by SEM and EDS.

No.	Group *	Treatment	No. of Samples
1	Control—dentin	No treatment	3
2	CoJet—sand	Air abrasion of dentin	3
3	Sand + etching	Air abrasion + Scotchbond multi-purpose etchant	3
4	Total-etch adhesive (TE)	Air abrasion + Scotchbond multi-purpose etchant + primer + adhesive	3
5	Self-etch adhesive (SE)	Air abrasion + Clearfil SE primer + adhesive	3

* All groups except the control were air abraded, as previously described.

**Table 4:** Results of the shear bond strength (MPa) test.

Group	Mean SBS (MPa)	Standard Deviation	Maximum	Minimum
Clearfil SE +SB	22.58	6.41	36.5	11.92
Clearfil SE −SB	21.33	5.05	30.62	10.35
Scotchbond +SB	17.48	6.75	33.42	7.96
Scotchbond −SB	14.33	6.27	25.74	4.59

+SB: with air abrasion; −SB: without air abrasion.

**Table 5:** Results of the shear bond strength test with Weibull parameters.

Treatment	Shape Parameter (β)	β 95%CI	Scale Parameter (ή)	ή 95% CI	r^2^
CLSE	4.9 (0.8)	3.56–6.75 a	23.27 (1.04)	19.11 (1.55) a	0.99
CLSE + SB	3.86 (0.61)	2.84–5.26 a,b	23.27 (1.04)	22.31–27.92 a	0.96
SCMP	2.32 (0.37)	1.69–3.17 b	15.68 (1.46)	13.07–18.82 b	0.95
SCMP + SB	2.65 (0.42)	1.93–3.62 a,b	19.11 (1.55)	16.29–22.41 a	0.96

CLSE, Clearfil SE; CLSE + SB, Clearfil SE + SB; SCMP, Scotchbond Multi-Purpose; CMP + SB, Scotchbond MP + SB; Same letters (a and b) indicate β and η values with no statistically significant differences (*p* > 0.05).

## Data Availability

Not applicable.
